# Joint Minimization of Uplink and Downlink Whole-Body Exposure Dose in Indoor Wireless Networks

**DOI:** 10.1155/2015/943415

**Published:** 2015-02-22

**Authors:** D. Plets, W. Joseph, K. Vanhecke, G. Vermeeren, J. Wiart, S. Aerts, N. Varsier, L. Martens

**Affiliations:** ^1^Information Technology Department, Ghent University/iMinds, Gaston Crommenlaan 8, 9050 Ghent, Belgium; ^2^Orange Labs Networks and Carriers, 38-40 rue Général Leclerc, 92794 Issy Les Moulineaux, France

## Abstract

The total whole-body exposure dose in indoor wireless networks is minimized. For the first time, indoor wireless networks are designed and simulated for a minimal exposure dose, where both uplink and downlink are considered. The impact of the minimization is numerically assessed for four scenarios: two WiFi configurations with different throughputs, a Universal Mobile Telecommunications System (UMTS) configuration for phone call traffic, and a Long-Term Evolution (LTE) configuration with a high data rate. Also, the influence of the uplink usage on the total absorbed dose is characterized. Downlink dose reductions of at least 75% are observed when adding more base stations with a lower transmit power. Total dose reductions decrease with increasing uplink usage for WiFi due to the lack of uplink power control but are maintained for LTE and UMTS. Uplink doses become dominant over downlink doses for usages of only a few seconds for WiFi. For UMTS and LTE, an almost continuous uplink usage is required to have a significant effect on the total dose, thanks to the power control mechanism.

## 1. Introduction

The vast expansion of the use of wireless networks in everyday life has led to a greater awareness of exposure of the general public to RF (radio-frequency) electromagnetic fields used for wireless telecommunication. International organizations such as IEEE [1] and ICNIRP (International Commission on Non-Ionizing Radiation Protection) [2] have issued safety guidelines to limit the maximal electric-field strength due to wireless communications. Also, on a national level, authorities have implemented laws and norms to limit the exposure to electromagnetic fields. A lot of research has been done on the characterization of RF exposure (e.g., [3–7]), and measurements have indicated that exposure in indoor environments cannot be neglected [8].

Exposure studies mostly consider either the fields generated due to traffic from base station to user device (downlink) or exposure due to the electromagnetic waves induced in the body by the user device (uplink). Further, software tools for predicting the received signal quality [9–15] very often focus on Quality of Services parameters and do not account for exposure values. In [16], the authors presented the WiCa Heuristic Indoor Propagation Prediction (WHIPP) tool, a set of heuristic planning algorithms, experimentally validated for network planning in indoor environments [16]. The path loss prediction algorithm takes into account the effect of the environment on the wireless propagation channel and bases its calculations on the determination of the dominant path between transmitter and receiver, that is, the path along which the signal encounters the least obstruction. The WHIPP tool is designed for optimal network planning with a minimal number of access points (AP) [16]. In [17], this tool was extended for automatic network planning with limited or minimized downlink electric-field strength in indoor wireless networks, without impairing coverage. In [18], it was further extended with prediction algorithms to simulate and visualize electric-field strengths due to DL traffic and localized Specific Absorption Rate values in 10 g tissue (SAR_10 g_) due to UL traffic.

In this paper, instead of separating between UL (due to the mobile device's transmitted signal) and DL (due to the electric-fields E originating from the base stations or APs) traffic, exposure is spatially calculated as a whole-body dose due to both UL and DL [19]. Different optimization scenarios will be simulated using the WHIPP tool, and the impact on the whole-body dose will be numerically assessed. Additionally, the impact of the actual usage time on the dose reduction will be investigated. To the authors' knowledge, no indoor wireless network design solutions are yet available for the minimization of the total whole-body doses, where UL and DL contributions are both considered. Further, the impact of such redesign on the total exposure dose has not been quantified before, neither at specific locations in the building, nor globally over the entire building. Four scenarios (using WiFi, Universal Mobile Telecommunications System (UMTS), or Long-Term Evolution (LTE)) will be defined to investigate the influence of the number of indoor base stations, power control, and uplink transmission duration on the exposure.

In Section 2, the WHIPP tool used for minimization and assessment of exposure doses is discussed, the minimization metric (whole-body dose) is mathematically formulated, and the simulation scenarios are presented. In Section 3, the results for the different scenarios are presented and the impact of optimizing the network topology on the total dose is assessed, as well as the influence of the uplink usage on the total dose. Finally, conclusions and future work are presented in Section 4.

## 2. Materials and Methods

### 2.1. WHIPP Prediction Tool

The WHIPP algorithm is a heuristic planning algorithm, developed and validated for the prediction of path loss in indoor environments [16]. It takes into account the effect of the environment on the wireless propagation channel and has been developed for the prediction of the path loss on a grid over an entire building floor or at specific locations. The spatial granularity of the prediction is determined by the density of the grid points on the building floor. The algorithm bases its calculations on the determination of the dominant path between transmitter and receiver, that is, the path along which the signal encounters the lowest obstruction. This approach is justified by the fact that more than 95% of the energy received is contained in only 2 or 3 paths [11]. The dominant path is determined with a multidimensional optimization algorithm that searches the lowest total path loss, consisting of a distance loss (taking into account the length of the propagation path), a cumulated wall loss (taking into account the walls penetrated along the propagation path), and an interaction loss (taking into account the propagation direction changes of the path, e.g., around corners). The performance of the model has been validated with a large set of measurements in various buildings [16]. In contrast to many existing tools, no tuning of the tool's parameters is performed for the validation. Excellent correspondence between measurements and predictions is obtained, even for other buildings and floors [16]. The WHIPP tool contains a user interface that was developed in collaboration with usability experts. This allows visualizing not only path loss, throughput, or electric-field values, but, based on the formulations presented in the next section, also power densities, (localized or whole-body) absorption values, and doses.

### 2.2. Minimization Metric: Whole-Body Exposure Dose

The aim of the paper is to minimize the median whole-body absorbed dose *D*_wb-total_^50^ (J/kg) [19, 20] over a given building floor where indoor base stations are installed. The total whole-body dose *D*_wb-total_ at a certain location in a building is calculated as the sum of the whole-body dose *D*_wb-DL_ (J/kg) due to downlink and the whole-body dose *D*_wb-UL_ (J/kg) due to the user device's uplink:

(1) $$\begin{array}{c} D_{\text{wb-total}}=D_{\text{wb-DL}}+D_{\text{wb-UL}}. \end{array}$$

In the following sections, it will be explained how the downlink and uplink whole-body exposure doses are calculated.

#### 2.2.1. Downlink Whole-Body Absorbed Dose *D*_wb-DL_

To calculate *D*_wb-DL_ (J/kg), the whole-body SAR SAR_wb-DL_ (W/kg) due to downlink is multiplied by *T*_total_ (s); the time duration of the exposure

(2) $$\begin{array}{c} D_{\text{wb-DL}}=T_{\text{total}}\cdot \text{SA}{\text{R}}_{\text{wb-DL}}. \end{array}$$

SAR_wb-DL_ accounts for the downlink exposure due to all base stations and is calculated as follows:

(3) $$\begin{array}{c} \text{SA}{\text{R}}_{\text{wb-DL}}=\underset{\text{B}{\text{S}}_{i}}{\sum }\left(S_{\text{B}{\text{S}}_{i}}\cdot \text{SA}{\text{R}}_{\text{RE}{\text{F}}_{\text{wb}}}^{\text{DL-B}{\text{S}}_{\text{RA}{\text{T}}_{i}}}\right), \end{array}$$

where *S*_BS_*i*__ (W/m^2^) is the received power density due to base station BS_*i*_ (WiFi AP, UMTS/LTE femtocell) and SAR_REF_wb__^DL-BS_RAT_*i*__^ (W/kg per W/m^2^) is the reference whole-body SAR (for 1 W/m^2^ of received power density) due to BS_*i*_ using a certain Radio Access Technology (RAT). Power densities from RATs using different frequencies will contribute to SAR_wb-DL_ according to the reference whole-body SAR for the RAT at that frequency. Therefore, (3) sums over the power densities from each of the base stations BS_*i*_. The power density *S*_BS_*i*__ (W/m^2^) is related to the electric-field strength as follows:

(4) $$\begin{array}{c} S_{\text{B}{\text{S}}_{i}}=\frac{E_{\text{B}{\text{S}}_{i}}^{2}}{Z_{0}}=\frac{E_{\text{B}{\text{S}}_{i}}^{2}}{120\cdot \pi }=\frac{E_{\text{B}{\text{S}}_{i}}^{2}}{377}, \end{array}$$

where *E*_BS_*i*__ (V/m) is the electric-field strength due to base station BS_*i*_, observed at the considered location and with an assumed duty cycle of 100%. *Z*_0_ is the free-space impedance, equal to 377 Ω. For WiFi, the actual duty cycle DC [-] of the traffic generated by BS_*i*_ [21] must also be accounted for, since it represents the relative transmission time of a signal. In WiFi, signals are not transmitted continuously and therefore the predicted power densities at 100% operation need to be multiplied by the duty cycle. For UMTS and LTE, the duty cycle is 100% for downlink. When accounting for the duty cycle, (4) can be rewritten as follows:

(5) $$\begin{array}{c} S_{\text{B}{\text{S}}_{i}}=\frac{E_{\text{B}{\text{S}}_{i}}^{2}\cdot \text{DC}}{377}. \end{array}$$

#### 2.2.2. Uplink Whole-Body Absorbed Dose *D*_wb-UL_

To calculate *D*_wb-UL_ (J/kg), the whole-body SAR SAR_wb-UL_ (W/kg) due to uplink is multiplied by *T*_usage_ (s), the time duration of the usage. *T*_usage_ is a value between 0 and *T*_total_:

(6) $$\begin{array}{c} D_{\text{wb-UL}}=T_{\text{usage}}\cdot \text{SA}{\text{R}}_{\text{wb-UL}}. \end{array}$$

SAR_wb-UL_ is the SAR due to the UL traffic from the mobile device towards base station BS_*c*_ it is connected to, using a certain RAT. It is calculated as follows:

(7) $$\begin{array}{c} \text{SA}{\text{R}}_{\text{wb-UL}}=P_{\text{B}{\text{S}}_{c}}^{\text{Tx}}\cdot \text{DC}\cdot \text{SA}{\text{R}}_{\text{RE}{\text{F}}_{\text{wb}}}^{\text{U}{\text{L}}_{\text{RAT}}}, \end{array}$$

where *P*_BS_*c*__^Tx^ (W) is the mobile device's power transmitted towards the base station BS_*c*_ it is connected to, DC [-] is again the WiFi duty cycle of the UL traffic, and SAR_REF_wb__^UL_RAT_^ (W/kg per W) is the reference whole-body SAR (for 1 W of transmitted power) due to the mobile device operating at RAT. For UMTS and LTE, the duty cycle is 100% for uplink.

In future research, also whole-body absorption due to the uplink transmission of* other* users will be accounted for.

#### 2.2.3. Input Parameters

The equations formulated above now allow calculating absorbed doses. However, some of the parameters are required as input or need to be calculated by the WHIPP tool.

(i) In (5), *E*_BS_*i*__ (V/m) (electric-field strength due to base station BS_*i*_) can be calculated by the WHIPP tool as described in [17, 18, 22], where a far-field conversion formula between path loss and electric-field strength is presented:
  (8) PLdB=139−EERP=1 kW(dBμV/m) +20·log10(f)(MHz),
 with PL (dB) as the path loss between the transmitter and a receiver at a certain location, *E*_ERP=1 kW_ (dB*μ*V/m) as the received field strength for an ERP (Effective Radiated Power) of 1 kW, and *f* (MHz) as the frequency.
 Using (8) and the identity
  (9) $$\begin{array}{c} E\left(\text{V}/\text{m}\right)=E_{\text{ERP}=1\;\text{kW}}\left(\text{V}/\text{m}\right)\cdot \sqrt{\text{ERP}\left(\text{kW}\right)}, \end{array}$$
 and knowing that for dipoles ERP (dBm) = EIRP (dBm) − 2.15, we obtain the following formula for the electric-field strength *E*_BS_*i*__ (dBV/m) at a certain location, as a function of the EIRP_BS_*i*__ (dBm) of the base station, the path loss, and the base station's frequency *f*_BS_*i*__ (MHz):
  (10) $$\begin{array}{c} E_{\text{B}{\text{S}}_{i}}\left(\text{dBV}/\text{m}\right)=\text{EIR}{\text{P}}_{\text{B}{\text{S}}_{i}}-43.15+20\cdot \text{lo}{\text{g}}_{10}\left(f_{\text{B}{\text{S}}_{i}}\right)-\text{PL} \end{array}$$
 or, with *E*_BS_*i*__ expressed in (V/m),
  (11) $$\begin{array}{c} E_{\text{B}{\text{S}}_{i}}\left(\text{V}/\text{m}\right)=10^{\left(\text{EIR}{\text{P}}_{\text{B}{\text{S}}_{i}}-43.15+20\cdot \text{lo}{\text{g}}_{10}(f_{\text{B}{\text{S}}_{i}})-\text{PL}\right)/20}. \end{array}$$
 PL (dB) is here predicted by the WHIPP tool [16].
(ii) The* duty cycle* (in (5) and (7)) depends on the type and amount of traffic over the air [21]. In the following sections, simplified duty cycle value assumptions will be made, depending on the considered network topology.
(iii) In (7), *P*_BS_*c*__^Tx^ (dBm) (mobile device's power transmitted towards the base station BS it is connected to) for phone call connections with a UMTS femtocell will be calculated with the WHIPP tool as described in [18]:
  (12) $$\begin{array}{c} P_{\text{B}{\text{S}}_{c}}^{\text{Tx}}=P_{\text{sens}}+\text{PL}, \end{array}$$
 where *P*_sens_ (dBm) is the sensitivity of the UMTS femtocell base station for maintaining a UMTS phone call, determined and validated at −110 dBm [18]. PL (dB) is again the path loss between transmitter and receiver locations, as calculated by the WHIPP tool [16]. Thanks to power control, *P*_BS_*c*__^Tx^ values will be lower for good connections with the base stations (low PL). The lower and upper limits for *P*_BS_*c*__^Tx^ values are −57 and 23 dBm, respectively (see also [23]).
 For LTE, *P*_BS_*c*__^Tx^ (dBm) will be modeled as described in [24, 25]:
  (13) $$\begin{array}{c} P_{\text{B}{\text{S}}_{c}}^{\text{Tx}}=P_{0\_\text{pusch}}+\alpha \text{PL}+10\log\left(M\right)+\delta , \end{array}$$
 with *P*_0_pusch_ as the required received power at the femtocell base station (FBS) over a bandwidth of one resource block, *α* as a path-loss compensation factor, *M* as the number of resource blocks being used, and *δ* as a network-controlled factor, reflecting power increase or decrease commands. *α* will be assumed equal to 1 and *δ* equal to zero. For the 20-MHz channel used in this paper, *M* is equal to 100. For the considered indoor environment, *P*_0_pusch_ is set equal to −96 dBm [25]. The lower and upper limits for *P*_BS_*c*__^Tx^ values are −40 and 23 dBm, respectively [24].
 For WiFi, no power control is used and a fixed value of 20 dBm for *P*_BS_*c*__^Tx^ is assumed.
(iv) The* reference SAR values* for 1 W/m^2^ observed power density from (3) and for 1 W transmitted power from (7) are listed in Table 1 for the three considered RATs. The downlink whole-body reference SAR values for UMTS and WiFi are obtained from [20]. As human model, the Duke model of the Virtual Family was used [26]. It is generated from a set of magnetic resonance images of whole-body scans from a 34-year-old male (height of 1.74 m, weight of 72 kg, and body mass index of 23.1 kg/m). Since the LTE downlink frequency band (2.6 GHz) is close to the WiFi band (2.4 GHz), it is fair to assume the same value for LTE as for WiFi. The uplink whole-body reference SAR values for UMTS are also obtained from [20] (cell phone placed to the right side of the head of the human model). The whole-body reference SAR value SAR_REF_wb__^UL_RAT_^ values for WiFi and LTE for data usage are obtained through Finite-Difference Time-Domain (FDTD) simulations, where the mobile device is held in front of the body. The resolution of the human model was chosen to be 2 mm × 2 mm × 2 mm, resulting in a total of about 110 million voxels [26]. The same mobile phone is assumed as in [18].

#### 2.2.4. Optimization Algorithm

In [17], an automatic electric-field minimization algorithm was designed, providing a user-defined throughput while ensuring a low and homogeneous field strength. It is based on the creation of networks consisting of low-power access points. This not only lowers the downlink exposure but, for technologies using power control mechanisms, also reduces uplink exposure thanks to the higher probability of access points being located near the user device. In the following, four scenarios will be defined to assess the impact of low-exposure indoor wireless deployments on the uplink, downlink, and total whole-body exposure doses. For each scenario, the wireless networks will be designed based on the receiver sensitivities corresponding to the envisioned throughput.

### 2.3. Scenarios

**Figure 1:**
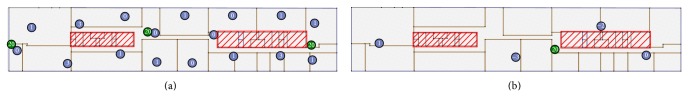
WiFi network configurations for the traditional deployment (green APs with EIRP = 20 dBm) and for the exposure-optimized configuration (purple APs with EIRP between −3 and 1 dBm) for (a) 54 Mbps and (b) 18 Mbps. EIRP in dBm is indicated within dots.

Two WiFi scenarios with different data requirements, one UMTS femtocell phone call scenario and one LTE femtocell data scenario, will be investigated. All scenarios are investigated in the office building depicted in Figure 1. The building is 90 m long and 17 m wide and consists of concrete walls (grey) and layered drywalls (brown). For all scenarios, a receiver height of 130 cm above ground level is assumed. For the design of the networks, a shadowing margin of 7 dB and a fading margin 5 dB are assumed [16].

#### 2.3.1. Scenario 1: WiFi Deployment for 54 Mbps: Device in front of Body

In the first scenario, two* WiFi* configurations are compared. The configurations are the ones from [17], in which a* traditional* network deployment (with maximal-power Equivalent Isotropically Radiated Powers (EIRPs)) and an* exposure-optimized* network deployment are compared based on only their electric-field strength distributions and based on a worst-case scenario with a duty cycle of 100%. The exposure-optimized scenario aims for a minimization of the median and the 95% percentile of the field values observed on the building floor [17]. Both deployments were designed to provide a high throughput (54 Mbps), corresponding to a receiver sensitivity of −68 dBm for an 802.11 b/g reference receiver [17].  Figure 1(a) shows the two network deployments. The traditional network deployment consists of the three 20 dBm APs (green) and the exposure-optimized deployment consists of the 17 low-EIRP APs (purple). The locations where no coverage is required (kitchen, toilet, shed, elevator, etc.) are shaded. For the downlink duty cycles, the assumption explained in [21] will be used. In the exposure-optimized configuration, the same amount of users is served by 5.66 (17/3) times more APs or one AP needs to serve 5.66 times less users. We assume that the downlink duty cycle for each AP is 3% for the exposure-optimized deployment and 17% (5.66 times 3%) for the traditional configuration. The uplink duty cycle will be assumed to be 2%, irrespective of the network configuration (duty cycles from [21]).

#### 2.3.2. Scenario 2: WiFi Deployment for 18 Mbps: Device in front of Body

In the second scenario, a traditional and an exposure-optimized deployment are designed for a throughput of 18 Mbps, corresponding to a receiver sensitivity of −82 dBm [17].  Figure 1(b) shows the two network deployments for scenario 2. The traditional network deployment consists of one 20 dBm green AP and the exposure-optimized deployment consists of the 4 low-EIRP purple APs. The locations where no coverage is required are again shaded. For the downlink duty cycles, the assumption explained in [21] will again be used. Compared to the traditional deployment in scenario 1, one AP is used instead of three and a duty cycle of 51% (3 times 17%) is assumed for the traditional deployment. The exposure-optimized deployment has four APs, so a duty cycle of 12.75% (51 divided by 4) is assumed. The uplink duty cycle will again be assumed to be 2%, irrespective of the network configuration (duty cycles from [21]).

#### 2.3.3. Scenario 3: UMTS Deployment for Voice Calls: Device to Side of Head

**Figure 2:**
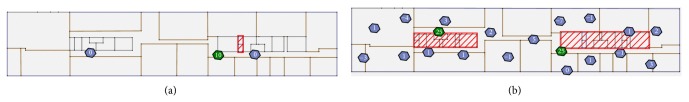
(a) UMTS network configurations for the traditional deployment (green FBSs with EIRP = 10 dBm) and for the exposure-optimized configuration (purple FBSs with EIRP of 0 dBm) for voice call connections and (b) LTE network configurations for the traditional deployment (green FBSs with EIRP = 25 dBm) and for the exposure-optimized configuration (purple FBSs with EIRP between −7 and 5 dBm) for 36 Mbps. EIRP in dBm is indicated within dots.

In the third scenario, a traditional and an exposure-optimized* UMTS* femtocell deployment are compared. The traditional deployment uses 1 UMTS femtocell base station (FBS) with an EIRP of 10 dBm to cover the entire building floor for* phone call coverage*, corresponding to a receiver sensitivity of −95.1 dBm [18]. The exposure-optimized deployment covers the floor with two UMTS FBSs with an EIRP of 0 dBm. In this UMTS scenario, the configuration with more FBSs (with a lower EIRP) will have the additional benefit of a lower average device transmitted power due to power control, whereas for WiFi the transmittance power of the mobile phone is fixed.  Figure 2 shows the two network deployments in the top figure. The traditional network deployment consists of one 10 dBm green FBS and the exposure-optimized deployment consists of two 0 dBm FBSs (purple). In the elevators, no phone call coverage is required. These areas are shaded.

#### 2.3.4. Scenario 4: LTE Deployment for 40.6 Mbps: Device in front of Body

In the fourth scenario, a traditional and an exposure-optimized* LTE* femtocell deployment for a throughput of 40.6 Mbps are designed. According to [17], for a 20 MHz channel, this corresponds to a required received power of −68.1 dBm [17].  Figure 2 shows the two network deployments in the bottom figure. In the traditional network deployment, the two green LTE FBSs with an EIRP of 25 dBm are sufficient to provide the required coverage, while the exposure-optimized deployment covers the floor with the 18 purple LTE FBSs with an EIRP between −7 and 5 dBm. In the shaded areas, no coverage is required. Exposure optimization clearly leads to a higher number of base stations and thus also to a higher cost.

## 3. Results and Discussion

### 3.1. Scenario 1: WiFi: 54 Mbps

**Figure 3:**
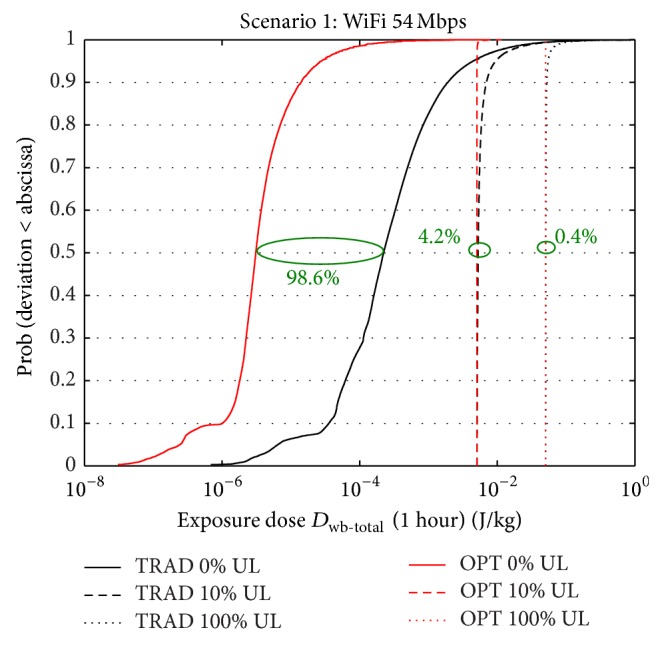
CDF of total dose (uplink + downlink) within one hour for scenario 1 for traditional and optimized deployment for three uplink usages (0%, 10%, and 100% of the time).

Figure 3 shows the cumulative distribution function (cdf) of the whole-body doses over a time frame of one hour for the traditional and optimized deployment for three uplink usages (0%, 10%, and 100% of the time) for WiFi with a downlink throughput of 54 Mbps. It also shows the median whole-body dose reductions when switching from a traditional to an exposure-optimized configuration. The mobile device is held in front of the body. The “0% UL” cdfs correspond with the DL-only dose.  Table 2 shows the 50% and 95% percentile of the doses for the different configurations of scenario 1. Figure 3 and Table 2 show that the downlink dose (0% UL) is drastically lowered in the exposure-optimized deployment (higher number of BS with a lower EIRP): a reduction of the median (*D*_wb-total_^50^), from 2.2 · 10^−4^ to 3.1 · 10^−6^ J/kg (reduction of 98.6%) and a reduction of 99.4% for the 95% percentile (*D*_wb-total_^95^) of the total dose. For the WiFi scenario, both configurations (traditional and optimized) will cause the same uplink powers and uplink doses due to the absence of power control in WiFi devices: irrespective of the connection quality with the AP, a fixed power of 20 dBm is assumed. Figure 3 and Table 2 show that, as the uplink usage increases (from 0 to 10 to 100%), the total dose is becoming quickly dominated by the uplink dose. For example, when comparing a usage of 10% with DL-only (0% usage), the median of the total dose (*D*_wb-total_^50^) is 24 times higher for the traditional deployment (0.0053 versus 2.22 · 10^−4^) and 1618 times higher for the optimized deployment (0.005 versus 3.09 · 10^−6^). The increasing dominance of the uplink causes the median dose reductions to become gradually smaller: from 98.6% for DL-only to 4.2% and 0.4% for 10% and 100% usage, respectively (see %RED in Table 2 and in Figure 3). The small dose reductions for an UL usage of 100% correspond to the almost coinciding plots for the traditional and optimized configurations. Unlike the median, the 95% percentile (highest doses) is still significantly reduced (46.6%) when using the optimized configuration with a 10% UL usage.

### 3.2. Scenario 2: WiFi: 18 Mbps

**Figure 4:**
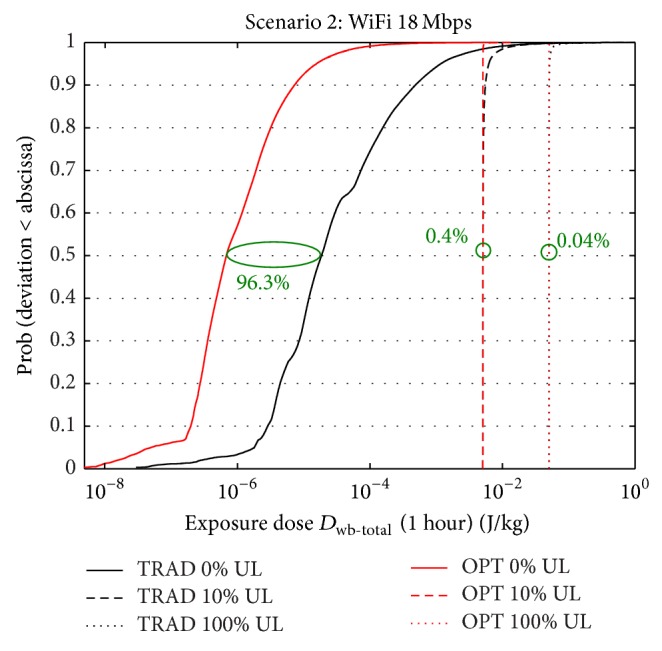
CDF of total dose (uplink + downlink) within one hour for scenario 2 for traditional and optimized deployment for three uplink usages (0%, 10%, and 100% of the time).

Figure 4 shows the cdf of the doses over a time frame of one hour for the traditional and optimized deployment for three uplink usages (0%, 10%, and 100% of the time), now for a WiFi throughput of 18 Mbps. It again shows the median whole-body dose reductions when switching from a traditional to an exposure-optimized configuration. Similarly, as in scenario 1, the downlink dose (0% UL) is drastically lowered when deploying more (lower-power) base stations (exposure-optimized) instead of fewer base stations with a higher EIRP (traditional): the reductions equal 96.3% and 98.7% for median and 95% percentile, respectively. Due to the lack of power control in WiFi, the total dose is again being dominated by the uplink dose for higher uplink usages, even more than that for scenario 1. When comparing a usage of 10% with DL-only (0% usage), the median of the total dose is 276 times higher for the traditional deployment (0.0051 versus 1.85 · 10^−5^ J/kg) and 7385 times higher for the optimized deployment (0.005 versus 6.77 · 10^−7^ J/kg), indicating that the DL dose quickly becomes negligible compared to the UL dose. Thanks to the lower throughput requirement, the DL doses are lower than those for scenario 1 (Table 2, 0% UL, scenario 1 versus scenario 2), indeed causing the UL dose (which is the same as that for scenario 1) to become more dominant. This is reflected by the lower reductions for the optimized configuration, for example, 0.4% (median) and 18.6% (95% percentile) for 10% usage in scenario 2, compared to 4.2% (median) and 46.6% (95% percentile) in scenario 1 (see %RED in Table 2 and in Figure 4).

### 3.3. Scenario 3: UMTS: Voice Calls

**Figure 5:**
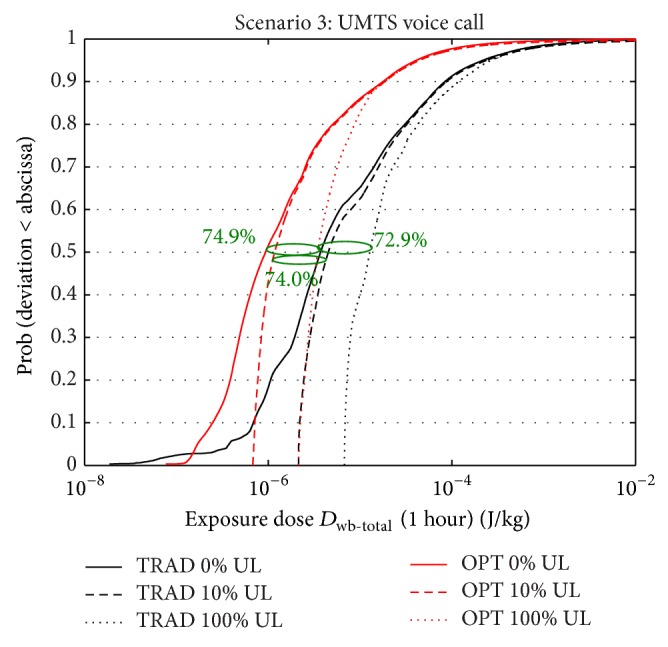
CDF of total dose (uplink + downlink) within one hour in an adult man for scenario 3 for traditional and optimized deployment for three uplink usages (0%, 10%, and 100% of the time).

Figure 5 shows the cdf of the doses over a time frame of one hour for the traditional and optimized deployment for three uplink usages (0%, 10%, and 100% of the time) for the UMTS voice call scenario, where the mobile device is held to the right side of the head. Again, a significant reduction is noticed in the total dose when deploying the optimized network. DL reductions are smaller than those for scenarios 1 and 2, due to the lower EIRP of the UMTS femtocell in the traditional deployment (10 dBm versus 20 dBm). However, total downlink doses are still reduced by 74.9% and 81.2% for median and 95% percentile, respectively (see %RED in Table 2 and Figure 5).

In addition to a lower DL dose and unlike for WiFi, the exposure-optimized deployment with more base stations also allows taking advantage of the power control mechanism in UMTS. Due to the higher number of base stations, the mobile phone will—on average—require a lower transmittance power to maintain its connection. Figure 5 and Table 2 show that, for increasing UL usages, the dose reductions are maintained. The reduction of the median dose for 10% and 100% UL usage is 74.0% and 72.9%, respectively, compared to 74.9% for DL-only. Also, for high UL usages, the UL dose does not become dominant over the DL dose: for the traditional deployment and for an uplink usage of 100%, the UL whole-body dose is only 2.4 times higher than the DL dose: 9.08 · 10^−6^ J/kg (“100% UL total dose” minus “0% UL total dose”) versus 3.72 · 10^−6^ J/kg. For the optimized deployment, the UL dose is 2.7 times higher. Figure 5 and Table 2 show that even when calling the entire time (100% UL usage), the whole-body total dose for the optimized deployment (3.46 · 10^−6^ J/kg) still remains below the whole-body total dose for the traditional deployment without UL usage (3.72 · 10^−6^ J/kg).

**Figure 6:**
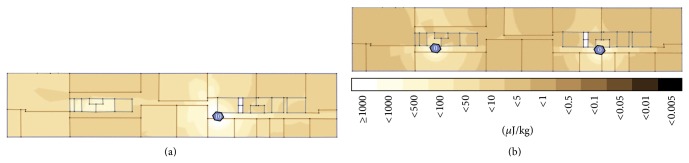
*D*_wb-total_ distribution for a one-hour period when calling the entire hour for traditional deployment (top) and optimized deployment (bottom). FBS EIRP in dBm is indicated within hexagon.

Figure 6 shows the total whole-body dose (in *μ*J/kg) in a one-hour period in an adult man (“Duke,” see Section 2.2.3) when calling the entire hour, for the two configurations: traditional (top) and optimized (bottom). The traditional deployment shows that the total dose is highly close to the FBS (high downlink dose) and near the cell edges (high uplink dose). For the optimized deployment, locations close to the FBS have lower downlink doses, thanks to the lower FBS EIRP, and locations far from the FBS have lower uplink doses thanks to the presence of the additional FBS. Figure 6 clearly shows that the high doses (lighter colors) are lowered for the optimized deployment.

### 3.4. Scenario 4: LTE: 40.6 Mbps

**Figure 7:**
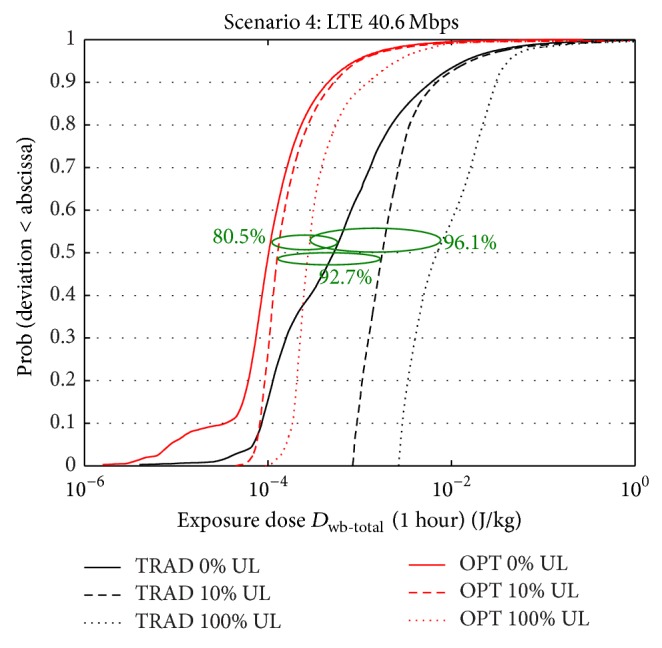
CDF of total dose (uplink + downlink) within one hour for scenario 4 for traditional and optimized deployment for three uplink usages (0%, 10%, and 100% of the time).

Figure 7 shows the cdf of the doses over a time frame of one hour for traditional and optimized deployment for three uplink usages (0%, 10%, and 100% of the time), for the LTE data scenario. The mobile device is again held in front of the body. Similarly to the previous scenarios, strong reductions of at least 80% are obtained, irrespective of the uplink usage. Analogously to UMTS, LTE also benefits from the power control mechanism. However, compared to UMTS, UL doses for LTE can become slightly more dominant over DL doses: for an uplink usage of 100%, the UL whole-body dose is 12 times higher than the DL dose for the traditional deployment (6.5 · 10^−3^ versus 5.21 · 10^−4^ J/kg) and 1.7 times for the optimized deployment (1.73 · 10^−4^ versus 1.02 · 10^−4^ J/kg). Indeed, for LTE, the optimized deployment is more beneficial for uplink dose reduction: as the UL usage increases, higher dose reductions are observed; for example, median dose reduction increases from 80.5% to 96.1% when uplink usage increases from 0% to 100%. For 100% uplink usage, the total dose for the optimized deployment (2.75 · 10^−5^ J/kg) is a factor 19 smaller than the total dose for the traditional deployment without UL usage (5.21 · 10^−4^ J/kg).

### 3.5. Comparison of Scenarios 1-2-3-4 for Varying Uplink Usages

**Figure 8:**
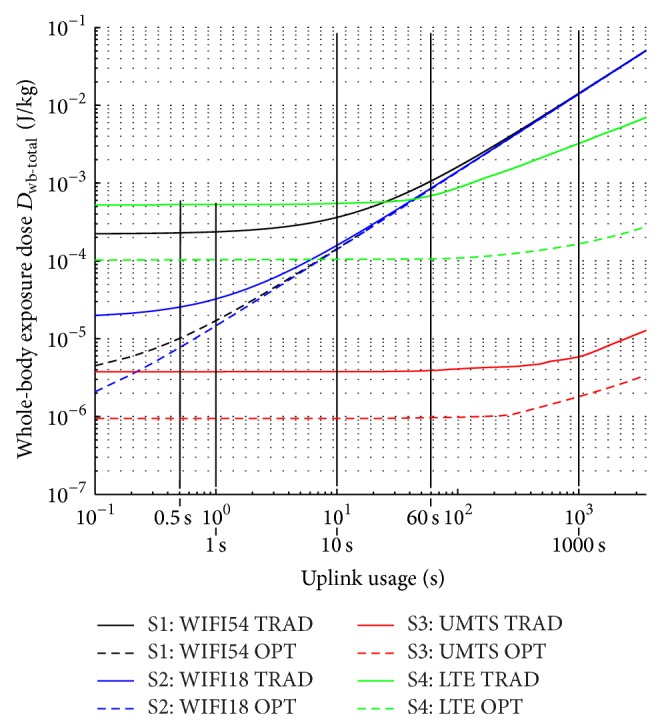
Whole-body total exposure dose within one hour as a function of uplink usage for the four scenarios and the traditional and optimized deployment.

Figure 8 compares the total median whole-body dose in the building as a function of the UL usage, ranging from 0 s to 3600 s (1 h) for all scenarios. It shows that, for no UL usage (0 s UL usage), scenario 4 has the highest exposure (due to the high-EIRP base stations), followed by scenario 1 (WiFi 54 Mbps), scenario 2 (WiFi 18 Mbps), and scenario 3 (UMTS). For the optimized WiFi deployments, the UL dose dominates the total dose for UL usages from less than 0.5 s already. For the traditional deployment for WiFi 18 Mbps, this occurs for UL usages from about 1 s and for the traditional deployment for WiFi 54 Mbps from about 10 s of UL usage.

Eventually, all WiFi scenarios converge to a total dose that is dominated by the same UL dose, due to the same uplink power of 20 dBm. For the traditional LTE deployment, UL dominates the total dose from about 60 s (1 min) of UL usage. For the optimized deployment, UL becomes dominant for usages around 1000 s (see logscale in Figure 8). Compared to WiFi, the total doses for LTE for high UL usages increase at a lower rate, thanks to the lower UL powers. This also counts for UMTS (scenario 3), which has the lowest doses, thanks to the lower throughput requirements and the power control mechanism.

All four scenarios show that significant exposure dose reductions can be achieved by adding more base stations with a lower transmit power. However, this comes at a higher economic cost. Future research consists of relating whole-body exposure dose due to the network to the total network installation cost [27].

## 4. Conclusions and Future Work

In this paper, total whole-body exposure doses (uplink and downlink) are jointly minimized for indoor wireless network deployments. The mathematical formulation has been given and four simulation scenarios were proposed: two WiFi configurations with a different throughput requirement, one UMTS voice call scenario, and one LTE high-throughput scenario. For WiFi, downlink doses are reduced by more than 95% by the optimized deployment. Due to the lack of power control, uplink usages of only a few seconds suffice to make the uplink dose higher than the downlink dose, limiting the reductions of the optimized deployment for longer uplink usages. Deployments with lower WiFi throughputs benefit less from optimizing the access point configuration. For UMTS, total dose reductions vary between 73% and 83%, irrespective of the uplink usage, thanks to the power control mechanism. For the LTE configuration with high-power base stations, dose reductions are at least 80% and increase for higher uplink usages. For UMTS and LTE, an almost continuous uplink usage is required to induce a significant effect on the total dose, again thanks to the power control mechanism.

In future research, the influence of the uplink transmission of other users will be accounted for and localized doses will be calculated. Also, a technoeconomic analysis will be done to link the (lower) exposure to the (higher) network installation cost, and the influence of the number of users and their usage profiles on the actual duty cycle of an access point will be investigated.

## Figures and Tables

**Table 1:** Reference whole-body SAR values SAR_REF_wb__^DL-BS_RAT_^ and SAR_REF_wb__^UL_RAT_^ for WiFi and UMTS, expressed in W/kg per 1 W transmitted power (UL) or in W/kg per 1 W/m^2^ observed power density (DL).

RAT	Frequency (MHz)	SAR_REF_wb__^DL-BS_RAT_^ (W/kg per W/m^2^)	SAR_REF_wb__^UL_RAT_^ (W/kg per W)
UMTS	1950	0.003	0.00495
WiFi	2400	0.0028	0.0070
LTE	2600	0.0028	0.0070

**Table 2:** 50% and 95% percentile values of total dose (J/kg) for four scenarios for traditional and optimized configurations for different uplink usages (0%, 10%, and 100%) and dose reduction (%). (TRAD = traditional deployment, OPT = optimized deployment, and %RED = exposure dose reduction percentage when switching from TRAD to OPT).

Total dose (J/kg)	Uplink usage								
	0% UL			10% UL			100% UL		
	TRAD	OPT	%RED	TRAD	OPT	%RED	TRAD	OPT	%RED
Scenario 1									
D_wb-total_^50^	2.22 · 10^−4^	3.09 · 10^−6^	98.6	0.0053	0.005	4.2	0.0506	0.0504	0.4
D_wb-total_^95^	0.0045	2.76 · 10^−5^	99.4	0.0095	0.0051	46.6	0.0549	0.0504	8.1
Scenario 2									
D_wb-total_^50^	1.85 · 10^−5^	6.77 · 10^−7^	96.3	0.0051	0.005	0.4	0.0504	0.0504	0.04
D_wb-total_^95^	0.0012	1.54 · 10^−5^	98.7	0.0062	0.0051	18.6	0.0516	0.0504	2.2
Scenario 3									
D_wb-total_^50^	3.72 · 10^−6^	9.35 · 10^−7^	74.9	4.53 · 10^−6^	1.18 · 10^−6^	74.0	1.28 · 10^−5^	3.46 · 10^−6^	72.9
D_wb-total_^95^	2.19 · 10^−4^	4.11 · 10^−5^	81.2	2.39 · 10^−4^	4.45 · 10^−5^	81.4	2.68 · 10^−4^	4.45 · 10^−5^	83.4
Scenario 4									
D_wb-total_^50^	5.21 · 10^−4^	1.02 · 10^−4^	80.5	1.80 · 10^−3^	1.28 · 10^−4^	92.7	7.00 · 10^−3^	2.75 · 10^−4^	96.1
D_wb-total_^95^	1.39 · 10^−2^	8.95 · 10^−4^	93.6	1.63 · 10^−2^	9.66 · 10^−4^	94.1	4.17 · 10^−2^	2.60 · 10^−3^	93.8
